# Consideration of the Psychological and Mental Health of the Elderly during COVID-19: A Theoretical Review

**DOI:** 10.3390/ijerph17218098

**Published:** 2020-11-03

**Authors:** Kunho Lee, Goo-Churl Jeong, JongEun Yim

**Affiliations:** 1 Department of Counselling Psychology, Sahmyook University, Seoul 01795, Korea; leekunho@syu.ac.kr (K.L.); gcjeong@syu.ac.kr (G.-C.J.); 2 Department of Physical Therapy, Sahmyook University, Seoul 01795, Korea; 3 Active Aging Research Institute, Sahmyook University, Seoul 01795, Korea

**Keywords:** COVID-19, elderly, pandemic, protective factors, mental health

## Abstract

COVID-19 has spread worldwide causing an unprecedented public health crisis. After the World Health Organization declared a pandemic in March 2020, the number of confirmed cases and deaths has continued to increase. This situation may be prolonged until an effective, tested, and safe treatment is available. COVID-19 can occur at any age. However, the maximum confirmed cases and deaths have occurred among the elderly. Particularly, the mental and physical health of the elderly aged above 60 and classified as high-risk groups is more vulnerable than other age groups, requiring more attention. Strong social restraint, social distancing, and quarantine measures to prevent the COVID-19 spread have raised concerns about their mental health. Therefore, it is crucial to analyze and identify the psychological concepts and protective factors that support and constitute these guidelines and strategies and prepare practical suggestions and guidelines to protect the mental health of the elderly during COVID-19. These discussions will facilitate a deeper understanding and expansion of these guidelines and strategies. Therefore, this study explores factors—including pandemic-induced stress, self-integration, self-efficacy, and resilience—in order to prepare practical and detailed suggestions and guidelines using studies that considered these factors, including coping with COVID-19-induced stress, social support, and physical activity.

## 1. Introduction

The world is suffering from changes caused by COVID-19. Given the argument that the new division of history should be pre- and post-COVID-19 [1], humanity is adapting to a new way of life in a new era to take preventive measures for nations and individuals.

Since the discovery of the first infected case in Wuhan, China, in December 2019, COVID-19 has spread beyond China and Asia and throughout the world, causing an unprecedented public health crisis. Shortly after declaring COVID-19 as a “public health emergency of international concern (PHEIC)” on 30 January 2020, the World Health Organization (WHO) declared it a “pandemic,” an infectious disease at its highest risk, on 11 March 2020 [2,3]. While the early spread in China and East Asian countries have recently slowed down to some degree, the number of confirmed cases and deaths in Europe and the United States has been rapidly increasing since the end of February. Moreover, because COVID-19 has spread to developing countries with relatively poor health conditions, inadequate public healthcare access and information dissemination, and limited (often sub-standard) medical infrastructure and available professional services, medical supplies, and proper treatment facilities (e.g., several countries in South America, Africa, and Asia among other regions), the pandemic is expected to linger for an unknown period until effective treatments are developed and the supply, distribution, and skilled application are achieved and stabilized over an appropriate time.

The widespread occurrence of infectious diseases, such as COVID-19, is closely related to symptoms of psychological distress, mental illness, and physical pain [4]. Furthermore, previous experiences of infectious diseases have shown that the number of people mentally affected by the pandemic exceeds the number of those physically infected by the disease, indicating the massive influence of such disease on mental health [5]. As demonstrated during (Middle East respiratory syndrome) MERS and severe acute respiratory syndrome (SARS), for example, pandemics had a significantly adverse effect on people’s mental health. Due to the outbreak of the (MERS) in 2015, countless citizens and patients experienced anxiety and fear [6]. The outbreak of severe acute respiratory syndrome (SARS) in Hong Kong in 2003 also caused mental health problems, such as stress, posttraumatic stress disorder (PTSD), and psychological distress [7]. According to a recent analysis of the psychology and mental state of Korean citizens affected by COVID-19, nearly half of the Korean population (48%), particularly 65% of the population in Daegu, where a mass outbreak had occurred, experienced depressive feelings due to the pandemic. The stress experienced by people due to COVID-19, compared to other disasters, was 1.5 times higher than that during MERS and 1.4 times higher than that caused by local earthquakes [8]. The harmful effects of COVID-19 on mental health are considered more extensive and powerful than those of prior epidemics, and consequently, national mental health is at serious risk. 

Another concern is that the prolonged pandemic situation may cause not only physical damage to individuals but also a collective form of intense stress [9]. Witnessing or experiencing a disaster causes mental shock, such as anxiety and depression, among individuals and spreads tension and fear like an infection and collectively affects the society. In cases of the Ebola virus, emotions such as fear and panic affected individuals and groups, increasing the occurrence of psychological distress and psychopathic symptoms [10]. Active treatment and intervention for national mental health have become an urgent need to the extent that psychological and mental quarantine, along with COVID-19 prevention, is a significant and increasingly more serious global concern. Furthermore, there is a need for a psychological support system for mental health and against future disasters caused by epidemics. 

## 2. Background Review

### 2.1. COVID-19 in the Elderly

As of 16 October 2020, there have been approximately 39,023,292 confirmed cases and 1,090,586 deaths worldwide [11]. Although COVID-19 can strike all age groups, notably most of the confirmed cases and deaths were found in the elderly [12]. According to a report by the US Centers for Disease Control and Prevention (CDC) in March 2020, more than 80% of deaths were found in patients aged >65 years, indicating vulnerability of the elderly to the virus [13,14]. Moreover, China has reported that the increase in severe infections and mortality rate of COVID-19 depends on age. Specifically, the incidence of severe infections in age groups 50–64, 65–79, and 80 years and above were approximately 19.8%, 43.2%, and 81.3% (*p* < 0.001), respectively, indicating a relation between the incidence of severe infections and age. Additionally, the mortality rate of the age groups increased to 1.2%, 4.5%, and 18.8% (*p* = 0.025), respectively [15]. In Korea, the average age of death was 75.7 years, and reports have shown that the fatality rate of COVID-19 increased with age [16]. Constitutionally, the elderly are vulnerable to serious infections and death due to reduced immune function and existing health conditions caused by aging [17,18]. Since 50% to 75% of Korean victims have underlying medical conditions, such as high blood pressure, diabetes, cardiovascular disease, chronic obstructive pulmonary disease, and cancer, they are more vulnerable to COVID-19 and are classified as the high-risk group [19,20]. Since the elderly, especially those with an underlying medical condition, are vulnerable to epidemics, health care, urgent intervention, and quarantine for the elderly become necessary. 

### 2.2. COVID-19 and Mental Health of the Elderly

The psychological and mental health issues caused by COVID-19 among the elderly should be discussed thoroughly and comprehensively. Particularly, more effort and attention is required for those aged above 60 years and are classified in the high-risk group [21] because they are physically and mentally more vulnerable than other age groups. A recent study conducted in China highlighted the negative effects of COVID-19 on individuals’ psychological and mental health. In a study conducted on the general public, 53.8% of respondents reported being psychologically affected at a moderate or severe level, with 16.5%, 28.8%, and 8.1% reporting symptoms of severe depression, anxiety, and stress, respectively [22]. Furthermore, 37.1% of the elderly had experienced depression and anxiety during the pandemic [23], and the emotional response of the elderly aged above 60 years was more apparent as compared to other age groups [24]. 

With recent signs of the prolongation of the pandemic, strong measures being implemented worldwide to prevent the spread of COVID-19, such as avoiding social activities, social distancing, and isolation, has further increased the mental health concerns among the elderly. Certainly, these social measures positively contribute to the effectiveness of disease prevention and the prevention of spread. However, the mental health of the elderly requires more attention and care because they are the demographic group who experience social isolation for the longest duration [25]. Additionally, as previous studies on the elderly have shown that social isolation is a “serious public health problem” [26] that increases the risk of cardiovascular, autoimmune, neurological, and mental health problems, the mental health problems of the elderly caused by COVID-19 should be more carefully discussed and addressed as a public health crisis. 

For a deeper understanding of the recent psychological and mental effects caused by the pandemic, emotions such as fear and anger should also be considered and observed [27]. Fear is a natural defense mechanism against potentially threatening events that requires greater attention because, when chronic or imbalanced, it can become a key component of various mental disorders [28]. During a pandemic, such as COVID-19, emotions such as fear and anger increase the rate of symptom manifestation and maintain high levels of anxiety and stress in vulnerable social groups, such as the elderly and those with existing mental disorders, as well as healthy people [29]. Moreover, past epidemics have shown that such conditions require more attention because they may cause depression, anxiety, PTSD, and mental illnesses and may even lead to suicide in severe cases [30,31]. In fact, during the SARS outbreak in 2003, there was an enormous increase in the suicide rate in the elderly [32]. 

The psychological epidemic of fear and panic caused by COVID-19 mushroomed more rapidly than the pandemic itself [8]. If COVID-19 penetrates the human body and paralyzes the lungs, the organ of life, the fear of the virus invades the brain, the control tower of the human body, and paralyzes reason. The fear of the virus extends to the entire population, not just those who are infected or worked directly with the infected. This also produces aversion and discrimination against patients or specific groups such as elderly, Chinese, and healthcare workers etc. Recently, the spread of fear and hatred against the elderly who are vulnerable to viruses have become serious social problems worldwide. Discriminatory behaviors, such as giving up providing treatment or mocking, towards the elderly have been occurring [33]. In these times, there is a desperate need to identify and/or devise proper interventions, strategies, and political measure to establish and maintain positive, healthy, and effective mental health conditions. Equally important is the concern over and communication with the elderly and various psychological and mental influences.

### 2.3. Considerations for Providing Psychological and Mental Health Guidelines for the Elderly during COVID-19

Efforts to protect mental health are as equally important as effort to physically prevent and treat COVID-19, especially among the elderly—the highest risk group. Thus, analyzing, proposing, and implementing strategies for practical psychological and mental treatment for the elderly is a priority relevant task. Many can be based on psychological support manuals for mental health suggested by international organizations, such as the World Health Organization (WHO), and disease control organizations such as the Center for Disease Control (CDC). 

Several psychological concepts and protective factors that support and compose the suggested guidelines and strategies should be addressed together while discussing the interventions for psychological and mental treatment during COVID-19. These concepts are essential to understand and further expand the suggested guidelines and strategies, which must be included to offset the negative psychological and mental influences and protect mental health. Moreover, discovering and conducting in-depth studies on new internal protective factors to aid mental health are necessary to further expand and develop these guidelines and strategies.

First, the discussion about COVID-19-induced stress is essential because the epidemic-induced stress [34] is the root of negative psychological and mental influences and, concurrently, a chief key concept that can offset those negative influences. According to recent studies conducted during the COVID-19 outbreak, stress not only caused psychological and mental distress and aggravated psychiatric symptoms but also led to suicide in severe cases [22,23,31,35]. Therefore, the right approach to initiate guidance and coping strategies against negative psychological and mental influences seems to be “coping with stress.” 

Second, a factor that must be addressed in this discussion is “ego-integrity.” Ego-integrity is the most ideal psychological state a person can have during advanced age [36] and includes self-worth and self-esteem [37]. Erikson stated that ego integrity among elderly is the state of psychological well-being, the ultimate state of not fearing death; that is, accepting one’s life without regret, being content with one’s life, and having a balanced view of the past, present, and future as a result of one’s adaptation to the skills and abilities required by the society, as one enters old age [38]. Constant exposure to high-stress levels in old age decreases expectations toward the self and damages positive self-perception [39]. In other words, high-stress levels, such as during COVID-19, in old age may negatively impact the development of ego-integrity. Furthermore, stress can lead to a loss of self-worth and depression [37], causing other mental health problems. Thus, ego-integrity is an essential factor that should be included in suggestions and strategies. Given that discriminatory behaviors, such as giving-up treatment or mocking, have become a social problem because of the growing aversions against the elderly due to COVID-19 paranoia, interventions based on their ego-integrity are particularly urgently needed. 

Third, another important factor is “self-efficacy.” “Self-efficacy” refers to the confidence or expectation of one’s ability to successfully perform an action or activity to produce desired results [40]. It is a major variable that predicts adaptation and plays an important role in behavioral change, physical health, mental problems, and psychological adaptation [41]. It may affect the degree to which the elderly can control and cope with events in stressful situations caused by COVID-19. According to previous studies, self-efficacy reduces the negative effects of stress [42,43], mediates 40% of the effects on depression [44], and plays a beneficial role in protecting and promoting physical and mental health in stressful situations [40]. Self-efficacy, therefore, not only helps the elderly to recognize their abilities in stressful situations caused by COVID-19 but also helps demonstrate the belief that they can overcome difficult situations. 

Finally, from the positive psychology perspective, “resilience” should receive attention. Resilience is defined as a positive strength to overcome and adapt to difficulties or stressful situations [45]. Importantly, high resilience among the elderly predicts variables such as high coping skills, long lifespan, low depression, a positive mind, strong social support networks, and dynamic physical activity; thus, resiliency is particularly important among the elderly where psychological, social, physical and socio-economic stresses are highest among all other age groups [46]. For these reasons, resilience is further defined as a protective factor that buffers and reduces risk factors that cause negative consequences [47]. Because resilience is expected to have a role and contribution as a protective mechanism for the elderly, it should be thoroughly examined with other psychological concepts (See Table 1).

Although each of the above factors may function independently in terms of their effect on psychological and mental health, they are interrelated and influence each other. Therefore, an integrated approach that acknowledges these relationships should be considered along with an individual approach. Furthermore, detailed and practical guidelines based on the studies of various psychological concepts and protective factors along with actions to aid the mental health of the elderly should be continuously studied. 

### 2.4. Suggestions for the Psychological and Mental Health of the Elderly during COVID-19

First, coping with pandemic-induced stress should be researched and discussed while developing new practical guidelines for the psychological and mental health specific to the elderly sector problems during the ongoing COVID-19 crisis. That is, research, discussions, and resulting guidelines should address elderly-specific problems, differing intensities of common problems the elderly experience, etc., while incorporating appropriate information, advice, coping mechanisms and long-term strategies in a comprehensive manner to include physical, social, cultural, psychological, mental, and other relevant dimensions. The stress caused by COVID-19 is a stressor that triggers psychological and mental distress in the elderly. An in-depth discussion on the matter is necessary because it is the beginning of psychological and mental problems and, concurrently, where the solution may be found. It is essential to initially consider the methodological problem of coping strategies to make suggestions for the psychological and mental health of the elderly during COVID-19. Although researchers generally classify coping strategies in various ways, the most effective method for handling stress caused by COVID-19 includes the appropriate and integrated use of problem-focused and emotion-focused coping strategies [48]. Although the two strategies do not have the same effect on managing stressful situations caused by the pandemic, they are considerably more effective when used together in a pandemic situation. That is, problem-focused coping eliminates the problems of stress in this unusual pandemic situation, while emotion-focused coping helps manage emotions accompanied by stress perception in this unavoidable circumstance. 

The following examples include some practical coping strategies applicable for daily life among the elderly (see also Table 2): (1) Stop reading, watching, and listening to news, including social media, about the pandemic because repeated exposure can cause stress and anxiety. (2) Refrain from spreading unofficial information. (3) Understand that it is normal to feel stress and fear in unpredictable situations. (4) Take deep breaths, stretch the body, and perform yoga or meditation. (5) Give attention to one’s needs, emotions, and thoughts. Monitor unpleasant emotions and clarify one’s thoughts. (6) Determine actions after considering collective and social influences. (7) Refrain from discriminating or blaming specific individuals or groups for the infection. (8) Take care of and encourage oneself. (9) Those with disabilities, such as mental illnesses or drug abuse, may be particularly vulnerable in an emergency, and thus, must continue with the treatment. Notice new symptoms or the worsening of existing symptoms, in which case, ask for medical help. 

Second, to protect the psychological and mental health of the elderly during the COVID-19 pandemic, the assurance of one’s ability to work with others, namely, social skills, should be considered. Such resources are related to social support, and as suggested by Lazarus and Folkman, is a personal resource that affects coping [49]. Considering the strong correlation between social support and lifespan attested through a study conducted in the Alameda district [50], social support is an important factor that can help the elderly maintain their health in a pandemic situation. More specifically, because social support convinces the elderly of their ability to control stressful situations, those with social networks tend to perceive stressors as less threatening than those who lack coping resources. Therefore, social support enables them to cope with pandemic-induced stress. Additionally, social support is closely related to various psychological protective factors, such as resilience [46]. Thus, it should be considered as the main factor for protecting the psychological and mental health of the elderly. 

Family relationships are a crucial part of social support for the elderly because it is an important environment that provides affection and protection. Hence, family support and relationships may be the solution for protecting the psychological and mental health of the elderly throughout the pandemic situation. Studies have shown that the elderly in low-functioning families with weak solidarity feel more depressed and lonely [51]. As with family, social engagement through religious activities may be another means for social support in stressful situations caused by the pandemic. The religious activity of the elderly is a mental resource that provides a frame of understanding about ordinary problems, such as sickness, death, and loss. This helps them become receptive to those problems [52]. It functions as an unofficial social support variable that helps the elderly integrate thoughts and overcome personal crises [53]. Because religious activity is closely related to ego-integrity and depression in the elderly, these factors should be considered together [54]. Therefore, the following examples include some coping strategies applicable in daily life for the elderly. (1) During the social distancing period, consider contacting family and friends through social media, phone calls, and the Internet. (2) Maintain regular religious activities and contact with the local community. Considering the pandemic situation, participate in religious activities, contact fellow believers, and maintain relationships with the local community through SNS and online. (3) Be informed, in advance, of where and how to receive counseling and other supporting services. (4) Notify close family and friends when symptoms of sadness, depression, and anxiety occur.

Third, physical activities should be considered for the psychological and mental health of the elderly. Regular physical activities play a positive role in preserving the functional capabilities of the elderly, prolonging independence in old age, reducing risk factors that cause medical disabilities, and maintaining the energy balance necessary for metabolism [55]. Additionally, physical activity is closely related to the mental well-being of the elderly, such as enhancing cognitive and emotional functions, influencing mental health and well-being by maintaining social networks, and improving their quality of life [56]. Previous studies on the relationship between physical activities and psychological factors among older adults have shown that regular physical activities help develop self-efficacy and bring changes in the perception of one’s health and happiness, thereby reducing depression [57,58,59]. Specifically, for the elderly, physical activities are naturally reduced due to restrictions on outside activities during the social distancing and quarantine period. The negative physical and mental influences affect their psychological, mental, and physical health. Therefore, there is significance in suggesting and developing regular and suitable physical activities for the psychological, mental, and physical health of the elderly during the pandemic situation. The following strategies and suggestions based on the above discussion are consistent with the recommendations of the WHO and CDC at national and local levels [34,60]. (1) Maintain a daily schedule and exercise pattern. (2) Have regular habits to maintain good health. (3) Make time for leisure activities and find enjoyable activities (e.g., indoor physical activities such as stretching, gymnastics, yoga, hula hoop, and dumbbell exercise). (4) Maintain a healthy and balanced diet. (5) Obtain enough sleep. (6) Avoid excessive drinking and drug use. (7) Take prescriptive medicine as usual (See Table 2).

## 3. Conclusions

The world is experiencing an unprecedented catastrophic situation due to COVID-19. Jones stated that in a disastrous situation, “a community whose people know and care about one another is the one that will pull through” [61]. In addition to acts to avoid infection, acts of concern, consideration, and respect for the most vulnerable social groups such as the elderly will be the sustaining power to overcome the COVID-19 crisis from beginning to end. This study examined the necessity and importance of the psychological and mental health of the elderly in the pandemic situation caused by COVID-19 and suggested several psychological factors and strategies for protecting mental health. The discussions presented in this study are useful as research resources for establishing guidelines to maintain psychological and mental health in the present pandemic situation and when preparing for future disasters caused by an epidemic. Moreover, the discussions will be useful for understanding and expanding guidelines and strategies for psychological and mental health. 

Through the discovery of new psychological internal protective factors and previous studies, this study is expected to promote further studies on the extraction and convergence of psychological protective factors to prepare for future disasters. 

Finally, we recommend the following for future studies. First, there is a need to develop psychological support services and vaccine programs that meet the specific needs of districts, ages, and groups, and studies on the introduction of a continuous psychological management system. Second, studies on prevention strategies for the increasing suicide rate in the post-COVID-19 economic crisis are needed. Third, to prevent the paralysis of the healthcare system in pandemic situations, it is necessary to conduct studies on the establishment of a response system for epidemic-psychiatric emergencies. Lastly, studies on facilities where psychiatric emergencies and epidemics can be simultaneously mediated in a pandemic situation are needed.

## Figures and Tables

**Table 1:** Considerations of Psychological Concepts and Influences during COVID-19.

Psychological Concepts	Psychological and Mental Influences
Stress	Stress not only causes psychological and mental distress and aggravates psychiatric symptoms, but may also lead to suicide in severe cases, according to recent studies conducted during the COVID-19 outbreak.
Ego-integrity	Constant exposure to high stress levels, such as those caused by COVID-19 among the elderly decreases expectations toward the self, damages positive self-perception, and can lead to loss of self-worth and depression; further contributing to or even causing other mental health problems.
Self-efficacy	Self-efficacy is a major variable that predicts adaptation and plays an important role in behavioral change, physical health, mental problems, and psychological adaptation. This may affect the degree to which the elderly can control and cope with events in stressful situations caused by COVID-19.
Resilience	High resilience among the elderly predicts variables such as high coping skills, long lifespan, low depression, a positive mind, strong social support networks, and dynamic physical activity; thus, resiliency is particularly important among the elderly where psychological, social, physical, and socio-economic stresses are highest among all age groups, especially during COVID-19.

**Table 2:** Practical Suggestions for the psychological and mental health of the elderly during COVID-19.

Considerations	Practical Suggestions
(Psychological Health) Stress caused by COVID-19	(1) Stop reading, watching, and listening to news, including social media, about the pandemic because repeated exposure can cause stress and anxiety. (2) Refrain from spreading unofficial information. (3) Understand that it is normal to feel stress and fear in unpredictable situations. (4) Take deep breaths, stretch the body, and perform yoga or meditation. (5) Give attention to one’s needs, emotions, and thoughts. (6) Determine actions after considering collective and social influences. (7) Refrain from discriminating or blaming specific individuals or groups for the infection. (8) Take care of and encourage oneself. (9) Those with disabilities, such as mental illnesses or drug abuse, may be particularly vulnerable in an emergency, and thus, must continue with the treatment. Notice new symptoms or the worsening of existing symptoms, in which case, ask for medical help.
(Social Health) Social support	(1) Maintain contact with family and friends. (2) Maintain regular religious activities and contact with the local community. (3) Be informed, in advance, of where and how to receive counseling and other supporting services. (4) Notify close family and friends when symptoms of sadness, depression, and anxiety occur.
(Physical Health) Physical activity	(1) Maintain a daily schedule and exercise pattern. (2) Have regular habits to maintain good health. (3) Make time for leisure activities and find enjoyable activities. (4) Maintain a healthy and balanced diet. (5) Obtain enough sleep. (6) Avoid excessive drinking and drug use. (7) Take prescriptive medicine as usual.
